# Retracted: Thyrotoxic Periodic Paralysis: Clinical Challenges

**DOI:** 10.1155/2021/7814159

**Published:** 2021-03-16

**Authors:** Journal of Thyroid Research

*Journal of Thyroid Research* has retracted the article titled “Thyrotoxic Periodic Paralysis: Clinical Challenges” [1]. The article was found to contain a substantial amount of material from previously published articles, including the following sources:

1. Shih-Hua Lin, Chou-Long Huang, “Mechanism of Thyrotoxic Periodic Paralysis,” JASN Jun 2012, 23 (6) 985–988; Doi: 10.1681/ASN.2012010046 [2].
2. Annie W. C. Kung. “Thyrotoxic Periodic Paralysis: A Diagnostic Challenge,” The Journal of Clinical Endocrinology and Metabolism, 2006, vol. 91 (7) 2490–2495 Doi: https://doi.org/10.1210/jc.2006-0356. [3].
3. Lam, L., Nair, R. J., and Tingle, L. “Thyrotoxic periodic paralysis”. Proceedings (Baylor University. Medical Center), 2006, 19 (2), 126–9. Doi: https://doi.org/10.1080/08998280.2006.11928143 [4].
4. C.-C. Chang, C.-J. Cheng, C.-C. Sung et al., “A 10-year analysis of thyrotoxic periodic paralysis in 135 patients: focus on symptomatology and precipitants,” European Journal of Endocrinology, vol. 169, no. 5, pp. 529–536. Doi: https://doi.org/10.1530/EJE-13-0381 [5]. (Cited as reference [24])
5. Oh, Sang Bo, Jinhee Ahn, Min Young Oh, Bo Gwang Choi, Ji Hyun Kang, Yun Kyung Jeon, Sang Soo Kim, Bo Hyun Kim, Yong Ki Kim, and In Joo Kim., “Thyrotoxic Periodic Paralysis Associated with Transient Thyrotoxicosis Due to Painless Thyroiditis,” Journal of Korean Medical Science, 2012 Doi: https://doi.org/10.3346/jkms.2012.27.7.822. [6].

The authors did not respond to our correspondence about this retraction, and it is therefore being retracted with the agreement of the editorial board.
